# Torsade de pointes and systemic azole antifungal agents: Analysis of global spontaneous safety reports

**DOI:** 10.21542/gcsp.2017.11

**Published:** 2017-06-30

**Authors:** M. Salem, T. Reichlin, D. Fasel, A. Leuppi-Taegtmeyer

**Affiliations:** 1 Department of Clinical Pharmacology and Toxicology, University & University Hospital Basel, Switzerland; 2 Department of Cardiology, University Hospital Basel, Switzerland; 3 Department of Infectious Diseases and Hospital Epidemiology, University Hospital Basel, Switzerland

## Abstract

**Background:** Literature about torsade de pointes induced by azole antifungal agents is scarce, despite the well-known association. Furthermore, little is known about the latency time between commencing an azole antifungal agent and developing torsade de pointes. The objectives of the present study were therefore to identify all cases of torsade de pointes associated with systemic azole antifungal use reported to the WHO monitoring centre (Uppsala, Sweden) and to determine the latency times between commencing the azole and developing torsade de pointes.

**Methods:** Investigator-driven, retrospective, descriptive analysis of post-marketing pharmacovigilance data regarding systemic azole antifungal agents and the development of torsade de pointes reported to the WHO monitoring centre 1995–2015.

**Results:** 191 cases were reported as follows: fluconazole 130, itraconazole 22, ketoconazole 5, posaconazole 1, voriconazole 33. More than half of all cases involved concomitant suspected or interacting drugs. The median latency times between starting the azole and developing torsade de pointes ranged from 1 (posaconazole) – 9.5 days (itraconazole), range <1–250).

**Conclusions:** Clinicians should be aware of these features of azole-associated torsade de pointes, avoid interacting drugs if at all possible and monitor at-risk patients.

## Background

Torsade de pointes is a special type of ventricular tachycardia which is preceded by the development of a long QT-interval in a patient’s electrocardiogram^1^. Torsade de pointes can be self-terminating causing dizzy spells and syncope, or it can degenerate into ventricular fibrillation, cardiac arrest and sudden cardiac death^1^.

Azole antifungal agents are known to cause QT-interval prolongation and in some cases subsequent torsade de pointes through inhibition of cardiac hERG (human ether receptor a go-go) potassium channels^2^. Literature about torsade de pointes induced by azole antifungal agents, however is scarce, despite this well-known association^3^. Furthermore, little is known about the latency time between commencing an azole antifungal agent and developing torsade de pointes.

The objectives of the present study were therefore to identify all cases of torsade de pointes associated with systemic azole antifungal use reported to the WHO monitoring centre (Uppsala, Sweden) and to determine the latency times between commencing the azole and developing torsade de pointes.

## Methods

An investigator-driven, retrospective, descriptive analysis of post-marketing pharmacovigilance data reported to the WHO monitoring centre 1995–2015 was performed. Data were available in the form of individual case safety reports (ICSRs) in the global database VigiBase™ which was accessed using the web-based platform Vigilyze™ (http://vigilyze.who-umc.org/. Accessed January 2016). The following drugs were searched for in conjunction with torsade de pointes: fluconazole, itraconazole, ketoconazole, posaconazole and voriconazole. The reaction term according to MedDRA (Medical Dictionary for Regulatory Activities) terminology was “Torsade de pointes”. The number of reports per drug, reporting countries, patient demographics and outcome were recorded. Where possible, the latency time between starting the drug and the development of torsade de pointes was also determined. As this data was not always available, the date of stopping the suspected drug was used as a surrogate for the date of occurrence of the reaction in some cases as it is usual clinical practice to withdraw a likely causative drug as soon as an adverse drug reaction is recognised.

## Results

**Figure 1.**
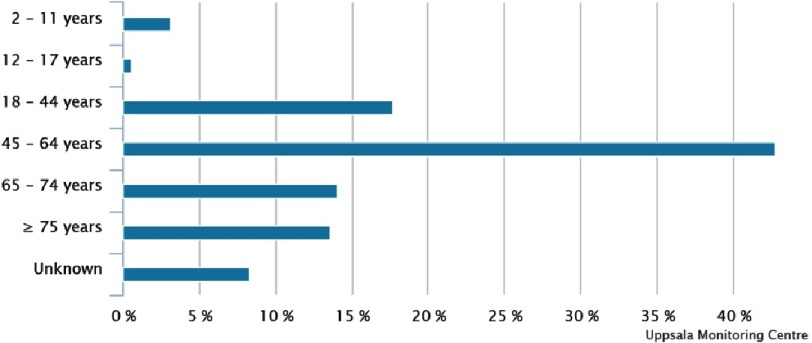
Age distribution of the 191 reported patients.

There were 191 reports of torsade de pointes in association with systemic azole antifungal use present in the WHO VigiBase™ database. The ICRs are summarised in Table 1 and Figure 1 shows the age distribution of the patients.

**Table 1:** Characteristics of individual case reports for systemic azole antifungal agents and torsades de pointes.

Drug	Number	Percent male, female, unknown (%)	Median age (years; range; number available)	Main reporting continent (%)
Fluconazole	130	46, 49, 5	60 (11–91; 119)	Americas (80)
Itraconazole	22	36, 59, 5	50 (24–92; 20)	Americas (45), Europe (45)
Ketoconazole	5	40, 60, 0	57 (37–75; 5)	Americas (80)
Posaconazole	1	0, 100, 0	15	Europe (100)
Voriconazole	33	53, 44, 3	57 (15–81; 30)	Americas (50)

**Figure 2.**
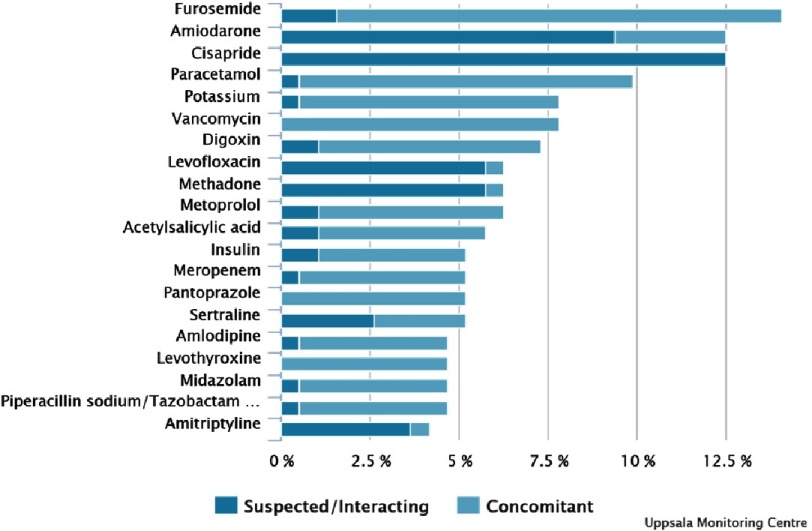
Top 20 co-reported substances (WHODrug).

Table 2 shows the data regarding the presence of co-suspected or interacting medication, latency time between starting azole antifungal agent and onset of reaction. Reported co-suspected or interacting medication included methadone, quinolone antibiotics, macrolide antibiotics, terfenadine, amiodarone and a number of different antiemetics (Figure 2 shows the top 20 co-reported substances). For itraconazole there were 2 cases where torsade de pointes occurred 7 and 4 days after stopping the drug, respectively. The single case where posaconazole was implicated had received voriconazole for 18 days prior to switching to posaconazole and then developing torsade de pointes within 24 hours.

**Table 2:** Presence of co-suspected or interacting medication, latency time and outcome data.

Drug	Cases with reported co-suspected or interacting medication (%)	Median latency time (days) (range; number available)	Percentage of cases with data available occurring within 1 day and 1 week	Reported outcome (recovered, death, unknown %)
Fluconazole	88 (68)	4 (<1–250; 52)	29, 59	25, 5, 70
Itraconazole	19 (86)	9.5 (1–26; 12)	8, 42	50, 5, 45
Ketoconazole	5 (100)	8 (1–219; 4)	25, 25	0, 20, 80
Posaconazole	1 (100)	1	100, 100	100, 0, 0
Voriconazole	17 (52)	8 (1–173; 23)	13, 48	27, 4, 69

## Discussion

This analysis of cases of torsade de pointes associated with systemic azole antifungal agents found that the majority of cases were reported from the Americas, that co-suspected and interacting drugs were present in the majority of cases and that latency time was during the first week in approximately half of all cases.

Geographic differences in reporting adverse drug reactions may reflect reporting habits of the different nations, the resources available for reporting or even genetic differences in predisposition to developing adverse drug reactions. Potential candidate genes for azole antifungal agents and the development of torsade de pointes are genes encoding drug-metabolising enzymes such as CYP2C19 and CYP2C9 which are involved in the metabolic clearance of azoles^3^ and genes encoding cardiac ion channels such as KCNH2^4^.

Concomitant treatment with other drugs known to be associated with QTc-prolongation and torsade de pointes or with drugs causing pharmacokinetic interactions leading to increased drug exposure are known risk factors for torsade de pointes^1–3^ and our study reconfirms that this is also true for systemic azole antifungal agents.

The latency time was found to be comparatively short in the majority of cases. This is in keeping with the nature of the adverse drug reaction which depends on drug binding to and inhibiting a receptor. This occurs rapidly after drug intake. A large longitudinal database study from The Netherlands also found that recent starters of non-cardiac QTc-prolonging agents were at increased risk of developing sudden cardiac death^5^. Schaffer and colleagues examined cases of torsade de pointes associated with macrolide use and found the mean time between commencing therapy and developing torsade de pointes to be 9 days (or 4 days if 3 outliers were excluded)^6^.

The fact that in some cases torsade de pointes occurred a number of days after stopping itraconazole can be explained by itraconazole’s long half-life of 42 hours, which may be increased further in the presence of hepatic impairment^7^.

A number of important drug-safety aspects for clinicians and their patients can be drawn from these findings. Firstly, prescribing azole antifungal agents in combination with interacting drugs should be avoided if at all possible. Secondly torsade de pointes can occur soon after commencing the drug and QTc-monitoring after commencing therapy may be prudent in high-risk patients. There are no established guidelines, but on the basis of currently available data, close monitoring during the first week would seem prudent, particularly for patients at high risk. Risk factors for QTc-prolongation include various drugs, metabolic disorders (i.e., hypokalemia), bradyarrhythmias, myocardial ischemia, congenital factors and other rare causes. Finally, torsade de pointes is associated with significant mortality.

Limitations of this study were that it is likely that not all cases of torsade de pointes present in the database were captured due to possible alternative coding such as “ventricular arrhythmia”, “ventricular tachycardia” or “cardiac arrest”. Furthermore, some cases may have been reported as being primarily due to another drug known to be associated with torsade de pointes with an azole classified as a concomitant, in which case they would not have been found by the search we performed. Pharmacovigilance data can also not deliver any information regarding the incidence of an adverse drug reaction because the total number of exposed people is not known. It is therefore not possible to determine which systemic azoles are more or less likely to cause torsade de pointes.

## Conclusions

Cases of torsade de pointes associated with systemic azole antifungal agents reported to the WHO during the past 20 years were mainly from the Americas, in the majority of cases co-suspected and interacting drugs were present and torsade de pointes occurred within one week of starting the drug in approximately half the cases. Clinicians should be aware of these features of azole-induced torsade de pointes and avoid interacting drugs if at all possible and monitor at-risk patients for the development of QTc-prolongation.

## Competing Interests

The authors have no competing interests to declare.

## Accompanying Statement

The data for this work were obtained from the WHO Collaborating Centre for International Drug Monitoring, Uppsala, Sweden. Data from spontaneous reporting are inhomogeneous as a result of different reporting policies worldwide and are vulnerable to underreporting and reporting bias. The information contained in this work comes from a variety of different sources and the likelihood that the suspected adverse reaction is drug-related is not the same in all cases. The information does not represent the opinion of the World Health Organization.

## Funding sources

No specific funding was received for this work.

## Authors’ contributions

All authors contributed to the conception and design of the study. MS and AT were involved in data acquisition and analysis. MS, DF, TR and AT interpreted the data, AT drafted the manuscript and MS, DF, TR and AT revised it critically for content. All authors read and approved the final manuscript.
